# Increased Incidence of Type 1 Diabetes during the COVID-19 Pandemic in Romanian Children

**DOI:** 10.3390/medicina57090973

**Published:** 2021-09-16

**Authors:** Adrian Vlad, Viorel Serban, Romulus Timar, Alexandra Sima, Veronica Botea, Oana Albai, Bogdan Timar, Mihaela Vlad

**Affiliations:** 1 Department of Internal Medicine II, Division of Diabetes, Nutrition and Metabolic Diseases, “Victor Babes” University of Medicine and Pharmacy, Eftimie Murgu Sq. no. 2, 300041 Timișoara, Romania; vlad.adrian@umft.ro (A.V.); timarrz@yahoo.com (R.T.); oana_olari@yahoo.com (O.A.); bogdan.timar@umft.ro (B.T.); 2 Center for Molecular Research in Nephrology and Vascular Disease, Faculty of Medicine, “Victor Babeș” University of Medicine and Pharmacy, Eftimie Murgu Sq. no. 2, 300041 Timișoara, Romania; vlad.mihaela@umft.ro; 3 “Cristian Serban” Medical Center for Evaluation and Rehabilitation for Children and Adolescents, str. Avram Iancu 18, 305100 Buzias, Romania; viorelseban@yahoo.com; 4 Center for Research in Preventive Medicine, Faculty of Medicine, “Victor Babeș” University of Medicine and Pharmacy, Eftimie Murgu Sq. no. 2, 300041 Timișoara, Romania; 5 Diabetes Outpatient Clinic, “Pius Brinzeu” Clinical County Emergency Hospital Timisoara, L. Rebreanu str. 156, 300723 Timisoara, Romania; veronicabotea@gmail.com; 6 Department of Internal Medicine II, Division of Endocrinology, “Victor Babes” University of Medicine and Pharmacy, Eftimie Murgu Sq. no. 2, 300041 Timișoara, Romania

**Keywords:** type 1 diabetes, COVID-19, incidence

## Abstract

*Background and Objective*: It is known that several viruses are involved in the pathogenesis of type 1 diabetes. Severe acute respiratory syndrome coronavirus 2 (SARS-CoV-2) is a new worldwide spread virus that may act as a trigger for the autoimmune destruction of the β-cells, as well, and thus lead to an increase in the incidence of type 1 diabetes. *Material and Methods:* The Romanian National Organization for the Protection of Children and Adolescents with Diabetes (ONROCAD) has collected information regarding new cases of type 1 diabetes in children aged 0 to 14 years from all over the country since 1996 and has computed the incidence of type 1 diabetes in this age group. *Results:* We observed a marked increase in the incidence of type 1 diabetes in the first year of the COVID-19 pandemic, with 16.9%, from 11.4/100,000 in 2019 to 13.3/100,000 in 2020, much higher compared to previous years (mean yearly increase was 5.1% in the period 1996–2015 and 0.8% in the interval 2015–2019). The proportion of newly diagnosed cases was significantly higher in the second half of 2020 compared to the second half of the previous years (57.8 vs. 51%, *p* < 0.0001). *Conclusions:* All these aspects suggest the role that SARS-CoV-2 could have in triggering pancreatic autoimmunity. To confirm this, however, collecting information from larger populations from different geographical regions, monitoring the incidence curves over a period of several years, and gathering background information on COVID-19 and/or data on COVID-19 specific antibodies are needed.

## 1. Introduction

It is well known that in most cases, type 1 diabetes is an autoimmune disorder. The theory regarding its pathogenesis states that in individuals with a permissive genetic background, various environmental factors (infectious, dietary, etc.) act as triggers for autoimmunity and lead, in time, to the partial or total destruction of the pancreatic β-cells and, as a consequence, to insulin deficiency. During the prediabetic period, as well as at the onset of the disease, some pancreatic antibodies may be detected in the patients’ serum, being markers of pancreatic autoimmunity [1]. Although this theory has a solid scientific background, until now it did not lead to the development of an effective method for the prevention of type 1 diabetes, despite numerous attempts.

Information regarding the epidemiology of type 1 diabetes may contribute to a better understanding of the pathogenesis of the disease. Worldwide, most of the published data refer to children aged 0 to 14 years, and they derive from national or regional diabetes registries, as well as from two international epidemiological studies, namely DIAMOND [2] and EURODIAB [3].

The role of some environmental factors as triggers for pancreatic autoimmunity is suggested by these epidemiological data. For example, vitamin D deficiency, which is common in regions close to the poles with few monthly sunshine hours, may explain the highest incidence of type 1 diabetes in these areas. The increasing exposure of the population to various food chemicals, as well as to viral infections, a consequence of increased mobility, may be the explanation for the almost constant increase in the incidence of type 1 diabetes in Europe, with an annual rate of 3.9%. Last but not least, the peak of the incidence of type 1 diabetes registered in the cold season can be explained by the involvement of seasonal viral infections [4,5]. There is plenty of information indicating the involvement of various viruses in the pathogenesis of type 1 diabetes: mumps and rubella virus, enteroviruses (Coxsackie B, Coxsackie A, Echo), cytomegalovirus, rotaviruses, retroviruses, etc. [6].

Severe acute respiratory syndrome coronavirus 2 (SARS-CoV-2), a positive-sense single-stranded RNA virus, is the etiologic agent for COVID-19, the illness responsible for the current pandemic. The virus exerts its effects after entering the human cells by binding to the angiotensin converting enzyme 2 (ACE2), an enzyme that lowers blood pressure by hydrolyzing angiotensin II into angiotensin 1-7, a peptide with vasodilating properties.

ACE2 is attached to the membrane of several cells, such as the enterocytes of the small bowel, the proximal tubular cells of the kidneys, the glandular cells of the gallbladder, the Sertoli and Leydig cells of the testis, the endothelial cells and pericytes of some blood vessels, the cardiomyocytes, the thyroid cells, the Langerhans islet cells, the hepatocytes, and cells from the placenta and the respiratory system [7]. Its expression in many tissues and organs explains the polymorphic symptoms of patients diagnosed with COVID-19.

It is known that between type 1 diabetes and COVID-19 there is a mutual influence: patients with diabetes have more aggressive forms of COVID-19, and the evolution of diabetes is more severe in the context of SARS-CoV-2 infection. Moreover, numerous cases of diabetic ketoacidosis have been reported, frequently as new onset [8]. To date, however, there is little information about the incidence of type 1 diabetes in the context of the pandemic [9,10], though its increase is very plausible given the immune implications of this viral infection.

## 2. Material and Methods

In Romania, a country where the medical care of children with diabetes is performed within a centralized system, the Romanian National Organization for the Protection of Children and Adolescents with Diabetes (ONROCAD) established the Romanian National Diabetes Register, starting in 1996. The register has been annually renewed thereafter, based on the data provided by the physicians managing children with diabetes. Over the years, several papers regarding the incidence of type 1 diabetes in children from this country have been published, showing that the incidence is growing steadily, at a faster pace than in other countries in Europe, and that there is a seasonality of new cases, the maximum incidence being registered in January, and the minimum in June [11,12,13].

Romania is a country severely affected by the COVID-19 pandemic. Thus far, more than 1 million cases have been diagnosed, of which more than 30,000 have died [14].

ONROCAD continued to obtain information on the incidence of type 1 diabetes in children after 2015, the year for which the latest data from children aged 0 to 14 years in Romania were published [13].

## 3. Results

The collected data showed that the incidence of type 1 diabetes in children tended to reach a plateau in the period 2015–2019, the average annual rate of increase being only 0.8%. In contrast, the incidence has increased very much, with 16.9% in 2020 compared to 2019 (Table 1).

The analysis of the distribution of new cases on a 6-months interval (January–June, respectively July–December) indicates that, in 2020, the proportion of cases diagnosed in the second half of the year (57.8%) was significantly higher (*p* < 0.0001) compared to previous years (51%).

## 4. Discussion

Our study indicates a marked increase in the incidence of type 1 diabetes in children aged 0 to 14 years in Romania in the first year of the COVID-19 pandemic, with 16.9%, an increase rate that is much higher compared to the previous years (5.1% in the period 1996–2015 and 0.8% in the interval 2015–2019) [13]. Furthermore, the proportion of newly diagnosed cases was significantly higher in the second half of 2020 compared to the second half of the previous years. Furthermore, in Romania there were numerous cases of COVID-19 in the last part of 2020, our country being only slightly affected by COVID-19 at the beginning of the pandemic [14], when a national lockdown was instituted. All these aspects suggest a putative role of SARS-CoV-2 in triggering pancreatic autoimmunity.

Our data suggest a correlation between the two aforementioned variables: the onset of type 1 diabetes mellitus, and COVID-19. Considering the accepted theory regarding the pathogenesis of type 1 diabetes [1], one can assume that SARS-CoV-2 could be a trigger for autoimmunity, and thus, a causality between the two diseases could exist. In order to demonstrate this causality, more information related to COVID-19 (history, antibodies) is required. However, taking into account that the purpose of collecting these data was to follow-up the evolution of the incidence of type 1 diabetes in children, information about COVID-19 is not available.

The data regarding a possible link between SARS-CoV-2 infection and new-onset type 1 diabetes in children is scarce and contradictory. One study, carried out in five centers from north west London during the peak of COVID-19 pandemic from 2020 [10], found an increase in incidence of 80% compared to a typical year, and, interestingly, the number of cases was higher in only two out of the five centers. Not all the children were submitted to tests that demonstrate the presence of current or past viral infection. The authors conclude that there is a possible link between SARS-CoV-2 infection and diabetes and recommend an increased awareness regarding this issue. Another study, performed in Germany [9], analyzed the incidence of childhood diabetes during the period of lockdown and found values within the predicted range (based on the evolution from the previous years). However, their data could be influenced by other factors, in addition to a SARS-CoV-2 infection, that might increase (psychological stress induced by lockdown) or decrease (reduced number of common viral infections that might trigger pancreatic autoimmunity because of social distancing) the incidence of new-onset type 1 diabetes. In addition, considering the existence of a variable delay between the action of the trigger and the onset of diabetes, in order to establish if the COVID-19 disease has a role in the pathogenesis of type 1 diabetes, a longer follow-up period is required.

Our paper has some weaknesses. First, the observation is made over a relatively short period of time (one year). In this situation, the marked increase in incidence in 2020 could be the result of statistical variability. Another bias could be that this significant increase in incidence will not be sustained over time, as it may be an earlier onset of type 1 diabetes due to the intervention of an additional trigger in patients who would have developed diabetes anyway. In order to clarify this issue, it is necessary to track the evolution of the incidence in the coming years. Second, there is no information on the existence of COVID-19 in the history of these cases, nor on the presence or absence of serum antibodies specific to this infectious disease. Finally, the information on new cases of diabetes is not verified by a second source. In previous publications, we have used as a secondary source the medical records of the patients admitted to the Clinical Center “Cristian Serban” in Buzias, which diagnoses and treats children and young people with type 1 diabetes from all over the country. Given the relatively short period elapsed since the diagnosis of the cases, as well as the pandemic limitation of hospitalizations in the center, this source could not be used at this point.

This paper also has several strengths. First of all, the information includes a large population, all the 19 million inhabitants of the country. Second, the medical care of children with type 1 diabetes is provided within a centralized system, involving a limited number of highly qualified physicians. This gives credibility to the diagnosis, on the one hand, and makes the collection of information quite easy and complete, on the other. Finally, the methodology used to collect information on new cases of type 1 diabetes in 2020 has been the same for over 20 years, namely direct contact with the pediatricians managing these children, being well developed and contributing to the reliability of the information.

## 5. Conclusions

Our incidence data suggest that SARS-CoV-2 might have a role in triggering type 1 diabetes. To confirm this, however, collecting information from larger populations from different geographical regions, monitoring the incidence curves over a period of several years, and gathering background information on COVID-19 and/or data on COVID-19 specific antibodies are needed.

## Figures and Tables

**Table 1:** Crude incidence rates for type 1 diabetes mellitus in Romanian children (0–14 years) between 2015 and 2020.

Year	Boys	Girls	Total
	Rate (/100,000)	Rate (/100,000)	Rate (/100,000)
2015	11.3	10.8	11
2016	11.6	13.2	12.3
2017	10.6	12.4	11.5
2018	11.1	11.3	11.2
2019	11.7	10.9	11.4
2020	13.5	13.0	13.3

## Data Availability

Data is available on request.
